# Acute high-dose diazepam reduces total bilirubin in bile: an experimental mouse study

**DOI:** 10.1007/s11419-026-00759-2

**Published:** 2026-03-03

**Authors:** Hiroshi Tsutsumi, Ako Sasao, Ryota Hiraiwa, Kyoko Hirata, Rie Sano

**Affiliations:** Department of Forensic Medicine, Faculty of Life Sciences, Kumamoto University, 1-1-1 Honjo, Chuo-ku, Kumamoto, 860-8556 Japan

**Keywords:** Diazepam, Bile, Bilirubin, Glucuronidation, Mouse

## Abstract

**Purpose:**

Acute drug intoxication may impair hepatic conjugation before overt liver failure. This study examined potential conjugation disturbances by assessing changes in serum and biliary bilirubin together with vital signs after an acute high dose of diazepam in mice.

**Methods:**

Male ICR mice (*n* = 9 per group) received an oral dose of diazepam (720 mg/kg) or vehicle (0.5% carboxymethylcellulose sodium) and were monitored for 120 min. Pulse rate, peripheral capillary oxygen saturation (SpO₂), and respiratory rate were recorded every 5 min using a pulse oximeter. At 120 min, the mice were euthanized by cervical dislocation, and blood and bile were collected. Total bilirubin concentrations in serum and bile were measured using a colorimetric assay with a lower limit of quantification of 0.16 mg/dL.

**Results:**

Diazepam-treated mice showed a greater decline in pulse rate than vehicle-treated mice, whereas SpO₂ and respiratory rate showed no significant between-group differences. Serum total bilirubin remained below the lower limit of quantification in all animals. By contrast, biliary total bilirubin concentrations were significantly lower in diazepam-treated mice than in vehicle controls (9.16 ± 0.76 vs. 5.31 ± 0.80 mg/dL, *p* = 0.01), despite comparable bile weight between groups.

**Conclusions:**

Acute high-dose diazepam selectively reduced biliary total bilirubin without marked hypoxemia or respiratory depression. These findings suggest that biliary bilirubin content may serve as a candidate marker of altered hepatic conjugation during acute diazepam intoxication and warrant further evaluation in analyses of human autopsy bile in forensic toxicology.

## Introduction

Drug-related morbidity and mortality have increased worldwide, with mental and substance use disorders representing a substantial component of the global burden of disease [1, 2]. Benzodiazepines are widely prescribed, and their involvement in both accidental and intentional overdoses has been reported in several countries [3, 4]. In forensic practice, benzodiazepine intoxication is typically diagnosed by integrating autopsy findings with postmortem toxicology, although interpretation often relies heavily on measured drug concentrations.

Conventional postmortem toxicology primarily relies on blood and urine, and conclusions are frequently drawn by comparing measured concentrations with reference ranges or reported lethal levels [5]. This approach can be problematic for rare compounds, novel psychoactive substances, or drugs with pronounced postmortem redistribution, and several case reports have highlighted difficulties in assigning cause of death when concentration data are limited or ambiguous [6–8]. There is therefore a need for complementary indicators that reflect toxicodynamic or pathophysiological changes rather than drug levels alone [9, 10].

Bile represents an attractive alternative matrix in forensic toxicology because it may contain higher concentrations of certain drugs and metabolites than blood, and it is relatively well preserved in decomposed bodies [11, 12]. At the same time, bile composition is influenced by hepatic transport and excretory mechanisms, and interpretation of postmortem bile drug concentrations remains challenging [13, 14]. Bilirubin is a canonical hepatobiliary analyte produced by heme catabolism, conjugated with glucuronic acid in hepatocytes, and excreted into bile [15]. Because glucuronidation is shared between bilirubin and many drugs, intensive drug metabolism could transiently alter bilirubin handling [16].

Diazepam is a long-acting benzodiazepine and GABA_A_ receptor agonist that undergoes extensive oxidative metabolism followed by glucuronidation [17, 18]. At high doses, diazepam produces sedation, muscle relaxation, and dose-dependent cardiorespiratory effects in experimental animals [19, 20]. We hypothesized that acute, high-dose diazepam administration may transiently perturb hepatic conjugation capacity and thereby modify biliary bilirubin content, even in the absence of overt hepatocellular injury or cholestasis.

This study therefore examines changes in total bilirubin in serum and bile, together with vital signs, in a mouse model of acute high-dose diazepam exposure. Our aim is to explore whether biliary bilirubin could serve as a candidate marker of altered hepatic conjugation during acute benzodiazepine intoxication and to provide a basis for future translational studies in forensic settings.

## Materials and methods

### Animals

Eighteen male ICR mice (10 weeks old; body weight 32–38 g) were purchased from Clea Japan, Inc. (Tokyo, Japan). The animals were housed in standard cages under controlled temperature and humidity, with a 12-h light–dark cycle and free access to standard chow and water. All experiments were conducted during the light period. Food was withdrawn 12 h before dosing, whereas water was provided ad libitum. The mice were randomly assigned to a diazepam group (*n* = 9) or a vehicle group (*n* = 9). All procedures were approved by the Animal Experiment Committee of Kumamoto University (approval number A 2025-083 R1) and were performed in accordance with institutional guidelines for animal care and use.

### Diazepam preparation and administration

Diazepam powder and carboxymethylcellulose sodium were purchased from FUJIFILM Wako Pure Chemical Corp. (Osaka, Japan). Diazepam was suspended in a 0.5% carboxymethylcellulose sodium solution immediately before use. The oral LD_50_ of diazepam in mice has been reported to be approximately 720 mg/kg in preclinical toxicity studies [21, 22]. Based on this information, mice in the diazepam group received a single oral dose of 720 mg/kg by gavage at a volume of 0.01 mL/g body weight. Control mice received an equal volume of 0.5% carboxymethylcellulose sodium (vehicle). After dosing, all animals were kept in the monitoring area for physiological measurements until euthanasia.

### Physiological monitoring

Pulse rate, peripheral capillary oxygen saturation (SpO₂), and respiratory rate were monitored using a MouseOx Plus pulse oximeter (Starr Life Sciences Corp., Oakmont, PA, USA). The sensor was attached to the neck of each mouse according to the manufacturer’s instructions. Following dosing, measurements were recorded at 5-min intervals for 120 min. The animals were visually observed throughout the monitoring period for signs of sedation, ataxia, or other abnormal behavior.

### Sample collection

At 120 min after dosing, the mice were euthanized by cervical dislocation. Whole blood was immediately collected by cardiac puncture into serum separator tubes and allowed to clot at room temperature before centrifugation. Serum was harvested and stored at 4 °C until analysis. The gallbladder was dissected, and bile was gently aspirated into pre-weighed microtubes. Bile weight was calculated as the difference between filled and empty tubes. Bile samples were stored at 4 °C, and all bilirubin measurements were completed within 24 h of collection.

### Bilirubin assays

Total bilirubin concentrations in serum and bile were quantified using the QuantiChrom Bilirubin Assay Kit (BioAssay Systems, Hayward, CA, USA) according to the manufacturer’s instructions. Bile samples were diluted 10-fold with a 5 mg/dL bovine serum albumin solution before measurement. The lower limit of quantification for bilirubin in this assay was 0.16 mg/dL. Because separation of conjugated and unconjugated fractions in bile was technically unreliable under the present conditions, only total bilirubin values were used for analysis.

### Statistical analysis

All data are presented as mean ± standard error of the mean. Bile weight and biliary total bilirubin concentrations were compared between the diazepam and vehicle groups using Welch’s *t*-test. Time-course data for pulse rate, SpO₂, and respiratory rate were analyzed using a mixed-effects model with fixed effects for time, treatment group, and their interaction. Statistical analyses were performed using GraphPad Prism 10 (GraphPad Software, San Diego, CA, USA). A two-sided *p* value < 0.05 was considered statistically significant.

## Results

### Baseline characteristics, bile collection, and clinical observations

All 18 mice completed the experimental protocol without intra-procedural complications. Body weight at the time of dosing did not differ systematically between the vehicle and diazepam groups (Table 1). Visual inspection confirmed that diazepam-treated mice developed marked sedation and reduced spontaneous activity, whereas vehicle-treated mice remained alert and mobile. No deaths occurred in either group during the 120-min observation period. Bile weight obtained at necropsy showed inter-individual variability but did not differ significantly between groups (Table 1).

**Table 1:** Baseline body weight and bile weight at necropsy in vehicle- and diazepam-treated mice (*n* = 9 per group)

Variable	Vehicle group (mean ± SEM)	Diazepam group (mean ± SEM)	*p*-value
*n*	9	9	-
Body weight (g)	35.1 ± 0.6	35.6 ± 0.7	0.63
Bile weight (mg)	21.1 ± 3.2	21.1 ± 2.5	1.00

SEM, standard error of the mean

Data are presented as mean ± SEM. Body weight was measured immediately before dosing, and bile weight was determined from gallbladder bile collected at necropsy. No statistically significant between-group differences were detected using Welch’s *t*-test

### Time course of vital signs

**Fig. 1:**
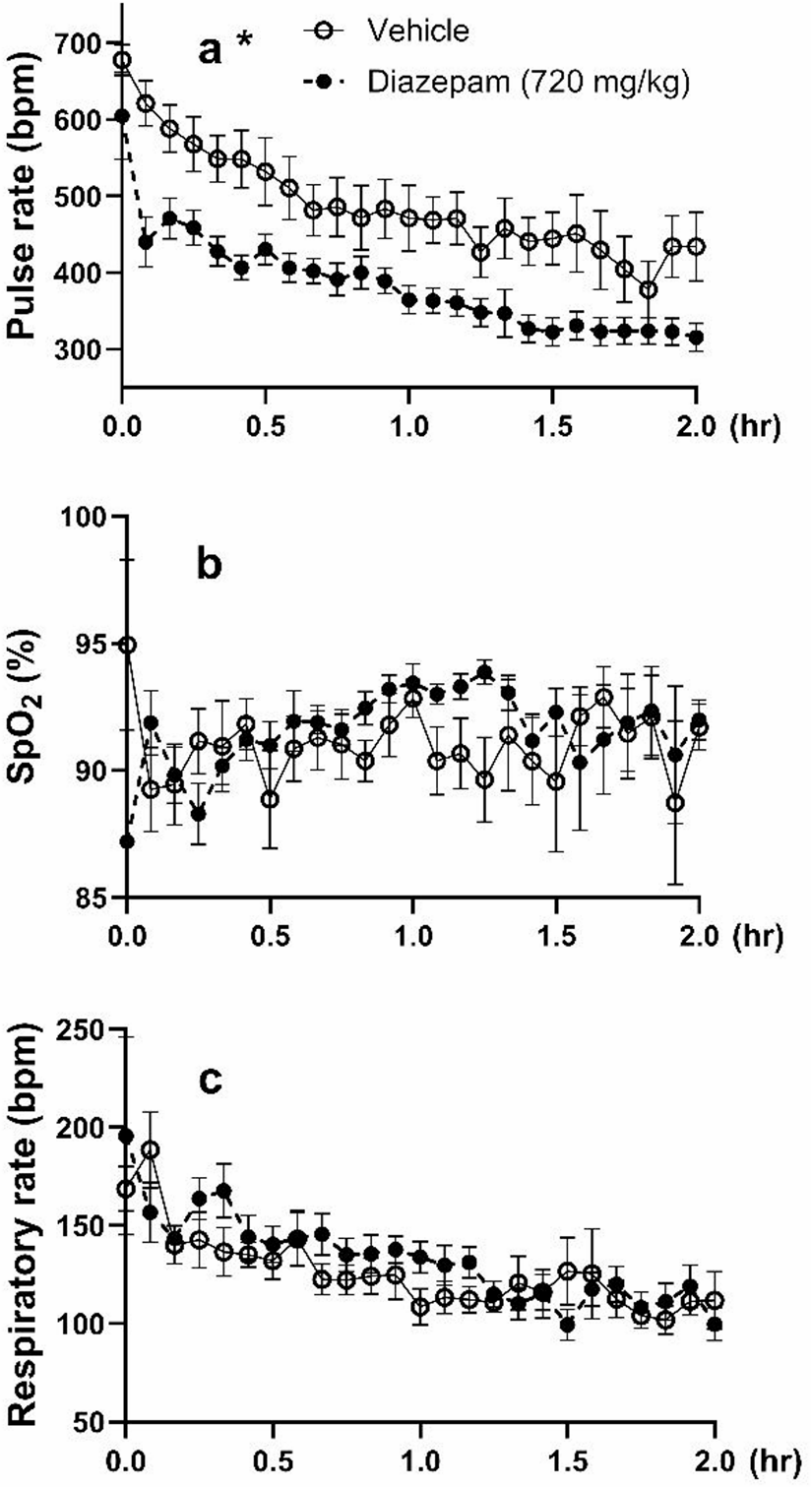
Time courses of vital signs after oral administration of vehicle or diazepam (720 mg/kg) in male ICR mice (*n* = 9 per group). (a) Pulse rate, (b) peripheral capillary oxygen saturation (SpO₂), and (c) respiratory rate during the 120-min monitoring period. Data are presented as mean ± standard error of the mean. * indicates a significant main effect of treatment on pulse rate in the mixed-effects model (*p* = 0.01)

Time courses of pulse rate, SpO₂, and respiratory rate are shown in Fig. 1a–c. Pulse rate declined over time in both groups and was lower overall in diazepam-treated mice than in vehicle controls (mixed-effects model: time effect, *p* < 0.01; treatment effect, *p* = 0.01; time × treatment interaction, *p* = 0.49) (Fig. 1a). SpO₂ fluctuated within a relatively narrow range (approximately 90%–94%) and did not differ significantly between groups (time effect, *p* = 0.52; treatment effect, *p* = 0.50; time × treatment interaction, *p* = 0.44) (Fig. 1b). Respiratory rate also gradually decreased over time in both groups, with no significant treatment effect (time effect, *p* < 0.01; treatment effect, *p* = 0.60; time × treatment interaction, *p* = 0.21) (Fig. 1c). These findings indicate that the selected diazepam dose produced pronounced bradycardia with only modest effects on oxygenation and respiratory rate.

### Serum and biliary bilirubin measurements

**Fig. 2:**
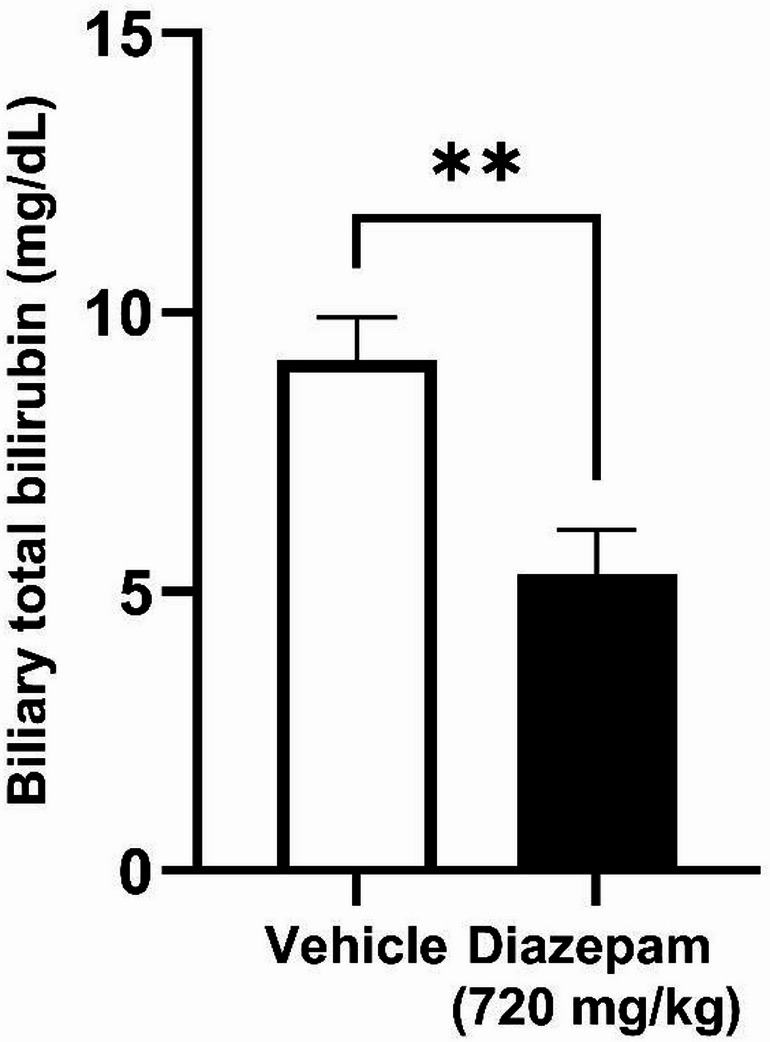
Biliary total bilirubin concentrations in vehicle- and diazepam-treated mice (*n* = 9 per group) at 120 min after dosing. Bars represent mean ± standard error of the mean. *p* = 0.01 (Welch’s *t*-test)

Serum total bilirubin concentrations were below the lower limit of quantification (0.16 mg/dL) in all mice, regardless of treatment. By contrast, biliary total bilirubin concentrations differed between groups. Vehicle-treated mice showed higher biliary total bilirubin levels (9.16 ± 0.76 mg/dL) than diazepam-treated mice (5.31 ± 0.80 mg/dL), and this difference was statistically significant (Welch’s *t*-test, *p* = 0.01) (Fig. 2). Thus, acute high-dose diazepam administration was associated with reduced biliary total bilirubin content under conditions in which bile weight was preserved.

## Discussion

This study describes an experimental mouse model in which an oral dose of diazepam close to the reported LD_50_ produced marked bradycardia and sedation without lethality or overt cholestasis, yet was associated with a significant reduction in biliary total bilirubin. This pattern is compatible with a specific alteration in hepatic conjugation and excretory processes during acute diazepam exposure, although the present study did not directly assess hepatic histology or global liver function.

One plausible mechanistic explanation is competition for hepatic glucuronidation capacity between diazepam (and its metabolites) and bilirubin. Bilirubin is conjugated predominantly by UDP-glucuronosyltransferase 1A1 (UGT1A1), and disturbances in this pathway can alter biliary bilirubin excretion without immediate changes in serum levels [15]. Many drugs, including benzodiazepines, undergo glucuronidation by overlapping UGT isoforms [16, 18]. Experimental and clinical data indicate that high substrate loads or co-administered drugs can saturate or competitively inhibit hepatic glucuronidation, leading to altered conjugation of endogenous substrates such as bilirubin [9]. In the present study, biliary total bilirubin decreased in diazepam-treated mice while serum bilirubin remained below the limit of quantification. A reduction in biliary bilirubin without detectable accumulation in serum suggests that conjugated bilirubin formation or canalicular transport may have been transiently constrained, but not to a degree sufficient to produce systemic hyperbilirubinemia. This pattern is compatible with the hypothesis that limited hepatic conjugation capacity was preferentially utilized for diazepam and its metabolites rather than for bilirubin. However, this mechanism should be regarded as diazepam-specific until comparable data are available for other GABA_A_ receptor agonists.

The vital sign changes observed in this study are consistent with the known pharmacological profile of high-dose diazepam. Preclinical studies have reported dose-dependent reductions in locomotor activity and cardiovascular depression following large diazepam doses in mice [19, 20]. Clinical literature on benzodiazepine poisoning similarly describes bradycardia and hypotension, particularly in severe cases or when combined with other central nervous system depressants [23, 24]. In the present model, diazepam produced a sustained decrease in pulse rate without major hypoxemia or respiratory depression (Fig. 1a–c), indicating a pharmacologically effective but non-lethal exposure. This context supports the interpretation that the observed reduction in biliary bilirubin occurred prior to the development of severe cardiorespiratory compromise.

Bile is increasingly recognized as a valuable but underused matrix in forensic toxicology, particularly in decomposed bodies or when blood is unavailable [11–14]. However, the toxicological significance of bile drug concentrations is difficult to interpret without reference to underlying hepatobiliary physiology. The present findings suggest that biliary bilirubin content may provide additional information on hepatic conjugation status in the setting of acute diazepam intoxication. Importantly, because bilirubin conjugation and biliary excretion can be altered by many conditions and xenobiotics, reduced biliary bilirubin should not be interpreted as a diazepam-specific indicator or a stand-alone basis for attributing exposure. Rather, if similar patterns are observed in human autopsy bile, biliary bilirubin may be considered a supportive, mechanism-oriented finding to be interpreted alongside comprehensive toxicological results, case circumstances, and pathological evaluation. At the same time, any such interpretation must account for pre-existing liver disease, cholestasis, and postmortem changes in bile composition.

This study has several limitations. First, the sample size was modest, and only male ICR mice were examined at a single, very high dose and at a single time point. Second, bilirubin concentrations in serum were below the limit of quantification, and bile volume was limited, which precluded reliable fractionation into conjugated and unconjugated bilirubin or simultaneous measurement of additional biliary constituents. Third, the study did not directly quantify diazepam or its metabolites in bile or liver, and therefore the proposed competition for glucuronidation remains inferential. Finally, extrapolation of these findings to humans or to other benzodiazepines is uncertain and will require systematic analysis of bile and related markers in human forensic autopsy cases, together with further studies in additional experimental models.

Despite these constraints, this model demonstrates that biliary total bilirubin can decrease measurably after acute high-dose diazepam administration in the absence of overt cholestasis or lethal toxicity. Future work should examine dose–response relationships, temporal dynamics, and the influence of underlying liver disease, and should include simultaneous measurement of hepatic and biliary diazepam metabolites. Ultimately, integrating bile-based indices of hepatic conjugation with conventional postmortem toxicology may help refine the interpretation of suspected benzodiazepine intoxication in forensic casework.

## Conclusions

Acute high-dose oral diazepam administration in mice produced marked bradycardia with minimal respiratory compromise and a significant reduction in biliary total bilirubin, while bile weight and serum bilirubin remained unchanged. These findings suggest that biliary bilirubin content may serve as a candidate indicator of altered hepatic conjugation during acute diazepam intoxication. Validation in human autopsy samples and across different dosing conditions will be essential before this approach can be adopted as a routine adjunct in forensic toxicology.

## Data Availability

The datasets generated and analyzed during the current study are available from the corresponding author on reasonable request.

## Declarations

### Use of AI-assisted tools

During preparation of this manuscript, the authors used ChatGPT (OpenAI) to assist with language editing and restructuring of the text. All suggestions from the tool were checked, edited, and approved by the authors, who take full responsibility for the content of the article.
